# Emergence of Human Rotavirus Group A Genotype G9 Strains, Wuhan, China

**DOI:** 10.3201/eid1310.070142

**Published:** 2007-10

**Authors:** Jihong Yang, Ting Wang, Yang Wang, Baojing Lu, Xuan Bai, Lei Zhang, Ming Wang, Hanzhong Wang

**Affiliations:** * State Key Laboratory of Virology WIV CAS, Wuhan, People’s Republic of China; † Central China Normal University, Wuhan, Hubei, People’s Republic of China; ‡ Xian Ning Center for Diseases Control and Prevention, Xian Ning, Hubei, People’s Republic of China

**Keywords:** Rotavirus, reverse transcription–PCR, G9, dispatch

## Abstract

Of 322 stool specimens collected from children with diarrhea from October 2005 through September 2006 in Wuhan, China, group A rotavirus was identified in 101 (31.4%). The most prevalent group A rotavirus genotype was G3P[8] (62.6%), followed by G1P[8](17.6%), G1+G3P[8](8.8%), G3P[4](6.6%), G1P[4](2.2%), and G9P[8](2.2%). The G9 strains were first detected in Wuhan.

## Abstract 2

Rotavirus Group A G9, China

Group A rotaviruses are the most common etiologic agents of severe diarrhea in infants and young children worldwide and are responsible for 2 million hospitalizations worldwide (*1**–**3*); in developing countries, these rotaviruses cause 400,000–500,000 deaths annually in children <5 years old. Previous molecular epidemiologic surveys have shown that 4 frequently observed genotype combinations—G1P[8], G2 P[4], G3 P[8], and G4 P[8]—are common worldwide (*3**,**4*) and, therefore, are prime candidates for current vaccine development strategies. Detailed epidemiologic studies have shown that rotavirus serotype G9 is emerging as an important human pathogen worldwide (*3*). Only 1 G9 strain was detected in Beijing between 1982 and 1997, However, G9 strains were identified in 3 regions in 1999; this increase coincided with the global trend. Our study involves the detection and characterization of G9 strains in Wuhan, a large city in central China; sequence analysis of the *vp*7 gene; and the phylogenetic analysis of the *vp*7 gene with that of G9 isolates in other regions of the world.

## The Study

A total of 322 stool specimens were collected from children with diarrhea who were hospitalized in 3 hospitals from October 2005 through September 2006 in Wuhan. Collection procedures were in accordance with rotavirus surveillance protocols recommended by the World Health Organization. Viral RNA was extracted from stool specimens with the guanidine isothiocyanate method as described (*5*). Rotavirus electropherotypes were determined by polyacrylamide gel electrophoresis according to the method described by Herring et al. (*6*)*.* The purified RNAs were examined by reverse transcription–PCR (RT–PCR) using the group A rotavirus–specific primers (Beg9/End9 and Con2/Con3) and then subjected to G genotype typing and P genotype typing by using a heminested RT–PCR strategy and electropherotyping (*5**,**7*). The result of G–P typing was confirmed by sequencing 10% of the total samples that were drawn randomly. Sequences of the RT–PCR products were analyzed with the Sequencher program (Gene Codes Corporation, Inc., Ann Arbor, MI, USA) and subsequently compared with the *vp*7 gene sequences of 11 G9 strains from the GenBank database using E-ClustalW (http://align.genome.jp); the phylogenetic tree was built by Molecular Evolutionary Genetics Analysis (MEGA) version 3.1 program (www.megasoftware.net).

Of 322 stool specimens, human group A rotavirus was detected in 101 (31.4%) case-patients. G typing of the 101 strains identified 67 as G3 (66.3%), 19 as G1 (18.8%), 8 as G1 and G3 mixed infection (7.9%), 4 as untypeable (4%), 1 as G4 (1%), and 2 as G9 (2%). Both rotavirus G and P types could be established in 91 strains, and 6 different combinations were identified (Table 1). G3P[8] (62.6%) was the most common combination detected, followed by G1P[8] (17.6%), G1/G3P[8] (8.8%), G3P[4] (6.6%), G1P[4] (2.2%), and G9P[8] (2.2%).

**Table 1:** Distribution of G and P genotypes of rotavirus strains from October 2005 to February 2006 in Wuhan, China

G type	P type	Total no. (%) rotavirus strains
G1	P[4]	2 (2.2)
G1	P[8]	16 (17.6)
G3	P[4]	6 (6.6)
G3	P[8]	57 (62.6)
G9	P[8]	2 (2.2)
Mixed G1,G3	P[8]	8 (8.8)
Total		91 (100)

**Figure 1:**
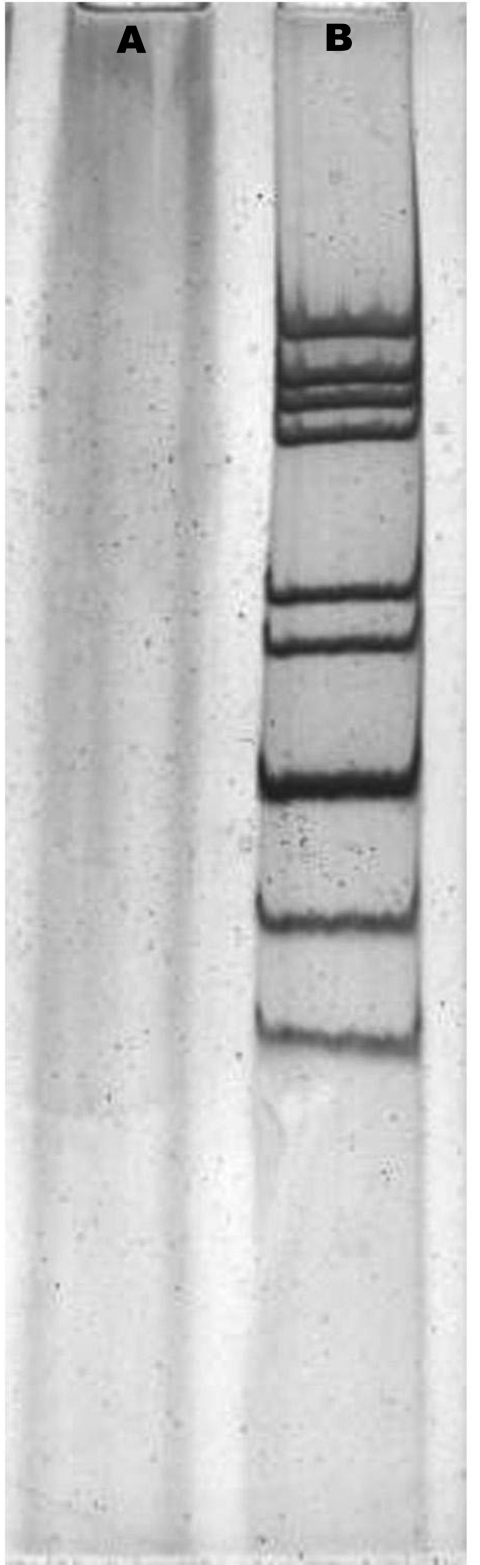
Polyacrylamide gel electrophoresis of rotavirus RNA. The viral RNAs were analyzed by electrophoresis in a polyacrylamide gel and visualized by silver staining. A, negative control; B, Wuhan G9 strain (CC589).

**Figure 2:**
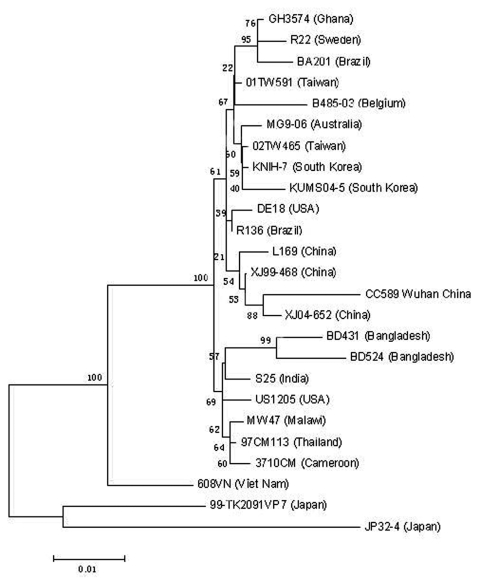
Phylogenetic analysis of the nucleotide sequences of the *vp*7 gene of G9 strain isolated in Wuhan and G9 strains from different parts of the world. The bar indicates the variation scale. A phylogenetic tree was generated based on the neighbor-joining method using MEGA version 3.1 (www.megasoftware.net). GenBank accession numbers of the *vp*7 gene sequences of group A rotavirus are the following: AY211068,AY196119,AY695811,DQ490173,AY307085,DQ096291,DQ990317,DQ056296, AY487877, AJ491163, AF438228, DQ321497, DQ321495, AJ250542, AJ250543, AJ491188, AF060487, AJ250544, AY866505, AY816184, AB091777, AB091756, AB176682, L79916, and EF197983.

Two group A rotavirus strains were designated to G9 genotype by electropherotypes (Figure 1) and RT–PCR. The complete nucleotide sequence and deduced amino acid sequence of the *vp7* gene from the Wuhan G9 strain were compared with the *vp*7 gene sequences of 11 G9 strains from the GenBank database (Table 2). The comparison showed clearly that the *vp*7 of the Wuhan G9 isolate was closely related to the 2 G9 strains (XJ99–468, XJ04–652) isolated from XinJiang province in China (98.3%–98.4% identity on nucleotide sequence and 97.2%–97.5% similarity on amino acid sequence). Phylogenetic analysis also confirmed that the *vp*7 gene of the Wuhan G9 strain (CC597, Wuhan, China) clustered in the same branch with those of the XJ99–468 and XJ04–652 G9 strains (Figure 2).

**Table 2:** Comparison of the nucleotide and amino acid sequence identity of *vp*7 gene of Wuhan G9 rotavirus strain (CC597) with those of 11 known G9 rotavirus strains

Strain	Similarity, %	
	Nucleotide	Amino acid
XJ04–652	98.4	97.5
XJ99–468	98.3	97.2
BD524	96.0	93.9
DE18	98.1	96.9
R136	98.0	96.6
KUMS04–5	98.2	96.0
02TW465	97.8	96.3
MG9–06	97.9	96.6
608VN	95.4	95.7
S25	97.6	96.6
JP32–4	90.7	92.3

## Conclusions

In this study, we presented the distribution of group A rotavirus genotypes and the emergence of the novel G9 genotype of rotavirus in Wuhan City from October 2005 through September 2006. Comparison with the results of the surveillance from 2000 to 2005 in Wuhan showed that the G3P[8] genotype remains the most predominant. Our results are similar to those reported by Fang et al (*8*), indicating that G3P[8] serotype was the most common strain throughout China from 2001 through 2003.

An important result of this study was the first characterization of the G9 strain isolated from Wuhan during the rotavirus season 2005–2006. Sequence analysis of the *vp*7 gene of the Wuhan G9 isolate showed that it had high similarity (97.5% amino acid identity) to that of the XJ04–652 strain, which was isolated from the specimen of a 9-day-old infant girl in XinJiang province, China, in December 2004 (Table 2). In the past decade, the number of countries that have reported the detection of rotavirus G9 strains has increased dramatically. Apparently G9 rotaviruses are expanding on a global scale; currently, the G9 serotype is considered to be the fifth most common type worldwide. In Australia, although the serotype G9 was identified for the first time in the 1999–2000 season, a retrospective analysis showed the presence of 3 G9 isolates in the country. During the 1999–2000 and 2000–2001 seasons, it rocketed to the second most common G type and was responsible for 10% and 18.1% of the total group A rotavirus infections in the country, respectively (*9*). In the 2001–2002 season, the G9 serotype was the most important infecting rotavirus serotype in the country, representing 40.4% of the isolates (*10*). In Spain, previous studies had identified G1P[8] and G4P[8] as the predominant cocirculating strains from 1995 through 2004. However, these serotypes were displaced by G9P[8] during the 2005 season, when G9P[8] accounted for 50.6% of typed isolates in several regions of Spain (*11*). Similarly, G9 serotype was identified as the prevailing serotype in several Japanese cities from 1998 to 2000 with high prevalence rates of 52.9% to 71.4% (*12*). In 1995, the rotavirus G9P[8] emerged in Bangladesh and became the second predominant strain (27.7%) during the 2001–2005 rotavirus seasons (*13*); G9P[8] accounted for most (91.6%) circulating rotavirus strains in Thailand during the 2000–2001 season and has now become the most common genotype (*14*). In China, G9 strains were identified in 3 regions including Kunming, Lanzhou, and Qinhuangdao in 1999 and 2003 (*8**,**15*); only 1 strain was detected between 1982 and 1997 in Beijing (*8*)*.* The incidence of G9 genotype increased from 0.9% to 4% from 1999 through July 2003, which coincided with an increased global incidence of G9 strain in various countries. However, it remains a mystery why G9 strain has only now been detected at low frequency in China because it has been one of the most prevalence rotavirus types worldwide for more than a decade.

Since the rotavirus G9 strain is the most widespread emerging rotavirus serotype globally, continued surveillance of rotavirus infections in China is imperative. Surveillance programs can effectively monitor whether G9 rotavirus strains will rise in prevalence. This information will be crucial for the development and evaluation of more efficient vaccines.

## References

[1] Parashar UD, Hummelman EG, Bresee JS, Miller MA, Glass RI. Global illness and deaths caused by rotavirus disease in children. Emerg Infect Dis. 2003;9:565–72. 12737740 10.3201/eid0905.020562 PMC2972763

[2] Parashar UD, Gibson CJ, Bresse JS, Glass RI. Rotavirus and severe childhood diarrhea. Emerg Infect Dis. 2006;12:304–6. 16494759 10.3201/eid1202.050006 PMC3373114

[3] Santos N, Hoshino Y. Global distribution of rotavirus serotypes/genotypes and its implication for the development and implementation of an effective rotavirus vaccine. Rev Med Virol. 2005;15:29–56. 10.1002/rmv.448 15484186

[4] Gentsch JR, Woods PA, Ramachandran M, Das BK, Leite JP, Alfieri A, Review of G and P typing results from a global collection of rotavirus strain: implications for vaccine development. J Infect Dis. 1996;1:30–6. 10.1093/infdis/174.supplement_1.s30 8752288

[5] Gentsch JR, Glass RI, Woods P, Gouvea V, Goeziglia M, Flores J, Identification of group A rotavirus gene 4 types by polymerase chain reaction. J Clin Microbiol. 1992;30:1365–73. 1320625 10.1128/jcm.30.6.1365-1373.1992 PMC265294

[6] Herring AJ, Inglis NF, Ojeh CK, Sondgrass DR. Rapid diagnosis of rotavirus infection by direct detection of viral nucleic acid in silver-stained polyacrylamide gels. J Clin Microbiol. 1982;16:473–517. 6182158 10.1128/jcm.16.3.473-477.1982 PMC272392

[7] Gouvea V, Glass RI, Woods P, Taniguchi K, Clark HF, Forrester B, Polymerase chain reaction amplification and typing of rotavirus nucleic acid from stool specimens. J Clin Microbiol. 1990;28:276–82. 2155916 10.1128/jcm.28.2.276-282.1990 PMC269590

[8] Fang ZY, Yang H, Qi J, Zhang J, Sun LW, Tang JY, Diversity of rotavirus strains among children with acute diarrhea in China: 1998–2000 surveillance study. J Clin Microbiol. 2002;40:1875–8. 10.1128/JCM.40.5.1875-1878.2002 11980983 PMC130922

[9] Masendycz P, Bogdanovic-Sakran N, Kirkwood C, Bishop R, Barnes G. Report of the Australia rotavirus surveillance program, 2000/2001. Commun Dis Intell. 2001;25:143–6. 11596718 10.33321/cdi.2001.25.31

[10] Kirkwood C, Bogdanovic-Sakran N, Clark R, Masendycz P, Bishop R, Barnes G. Report of the Australia rotavirus surveillance program, 2001/2002. Commun Dis Intell. 2002;26:537–40. 12549519 10.33321/cdi.2002.26.51

[11] Sánchez-Fauquier A, Montero V, Moreno S, Solé M, Colomina J, Iturriza-Gomara M, Human rotavirus G9 and G3 as major cause of diarrhea in hospitalized children, Spain. Emerg Infect Dis. 2006;12:1536–41. 17176568 10.3201/eid1210.060384 PMC3290946

[12] Zhou Y, Li L, Kim B, Kaneshi K, Nishimura S, Kuroiwa T, Rotavirus infection in children in Japan. Pediatr Int. 2000;42:428–39. 10.1046/j.1442-200x.2000.01247.x 10986883

[13] Rahman M, Sultana R, Ahmed G, Nahar S, Hassan ZM, Saiada F, Prevalence of G2P[4] and G12P[6] rotavirus, Bangladesh. Emerg Infect Dis. 2007;13:18–24. 10.3201/eid1301.060910 17370511 PMC2725799

[14] Khamrin P, Peerakome S, Wangsawasdi L, Tonusin S, Sornchai P, Maneerat V, Emergence of human G9 rotavirus with an exceptionally high frequency in children admitted to hospital with diarrhea in Chiang Mai, Thailand. J Med Virol. 2006;78:273–80. 10.1002/jmv.20536 16372282

[15] Fang ZY, Wang B, Kilgore PE, Bresee JS, Zhang LJ, Sun LW, Sentinel hospital surveillance for rotavirus diarrhea in the People’s Republic of China, August 2001–July 2003. J Infect Dis. 2005;192:S94–9. 10.1086/431505 16088812

